# Fully Untethered Battery-free Biomonitoring Electronic Tattoo with Wireless Energy Harvesting

**DOI:** 10.1038/s41598-020-62097-6

**Published:** 2020-03-26

**Authors:** José Alberto, Cristina Leal, Cláudio Fernandes, Pedro A. Lopes, Hugo Paisana, Aníbal T. de Almeida, Mahmoud Tavakoli

**Affiliations:** Institute of Systems and Robotics, Department of Electrical and Computer Engineering, University of Coimbra, Polo II, 3030-290 Coimbra, Portugal

**Keywords:** Electrical and electronic engineering, Sensors and biosensors, Biomedical engineering

## Abstract

Bioelectronics stickers that interface the human epidermis and collect electrophysiological data will constitute important tools in the future of healthcare. Rapid progress is enabled by novel fabrication methods for adhesive electronics patches that are soft, stretchable and conform to the human skin. Yet, the ultimate functionality of such systems still depends on rigid components such as silicon chips and the largest rigid component on these systems is usually the battery. In this work, we demonstrate a quickly deployable, untethered, battery-free, ultrathin (~5 μm) passive “electronic tattoo” that interfaces with the human skin for acquisition and transmission of physiological data. We show that the ultrathin film adapts well with the human skin, and allows an excellent signal to noise ratio, better than the gold-standard Ag/AgCl electrodes. To supply the required energy, we rely on a wireless power transfer (WPT) system, using a printed stretchable Ag-In-Ga coil, as well as printed biopotential acquisition electrodes. The tag is interfaced with data acquisition and communication electronics. This constitutes a “data-by-request” system. By approaching the scanning device to the applied tattoo, the patient’s electrophysiological data is read and stored to the caregiver device. The WPT device can provide more than 300 mW of measured power if it is transferred over the skin or 100 mW if it is implanted under the skin. As a case study, we transferred this temporary tattoo to the human skin and interfaced it with an electrocardiogram (ECG) device, which could send the volunteer’s heartbeat rate in real-time via Bluetooth.

## Introduction

Surface biopotentials collected from the human epidermis contain important information about human physiology, such as muscular, heart and brain activities. This includes electromyography (EMG)^1^, Electrocardiography (ECG)^2^, and Electroencephalography (EEG)^3^, among others. The collected data has applications in health monitoring (EMG, ECG, EEG), control of prosthetics^4^ or novel forms of wearable human-machine interfaces (EMG)^5,6^.

Wearable stickers that interface the human epidermis and acquire biopotentials for electrophysiological monitoring can be potentially transformative in digital health, since they would eventually allow a fully wireless and hassle-free data collection from the human body. Unlike traditional “wearable” technology that is composed of several rigid components, these stickers are required to be soft, flexible and stretchable. In this way, they are able to follow the dynamic morphology of the skin and remain attached to the skin during natural human movements. An ideal biomonitoring sticker is as well thin, imperceptible, comfortable and untethered. This can be also in the form of an electrical bandage or a “temporary tattoo” which bonds strongly to the human skin and acquires and transmits the information.

During the last five years, some reports on fabrication and applications of ultrathin stretchable electronic films, also called epidermal electronics^7^ or electronic tattoos have been presented^8–11^. The interest in E-tattoos is mainly because these films are comfortable and nearly imperceptible. Applications of these films have been shown in monitoring muscular activity through Electromyography (EMG) to control UAVs^12^ or prosthetic hands^13^, to monitor heart rate through Electrocardiography (ECG)^14,15^, for the acquisition of brain signals through Electroencephalography (EEG)^16^, and for electrochemical analysis of sweat^17^. While promising, one main limiting factor in the real-world application of these “tattoos” is the energy supply. Bulky rigid batteries, negatively affect the overall concept behind the electronic tattoos, i.e., their imperceptibility. While extensive efforts are made to make these stickers with sub-millimetre thickness, the current battery technology is hardly able to align itself with these requirements. An alternative approach that has been under research is the utilization of wireless power transfer (WPT) to power or charge biomedical devices^18–25^. These systems have the advantage of being able to transmit power to devices where it would be impossible to connect wires, as body implantable monitors^18^, nerve sensing or nerve stimulating systems^19^ or even reduce the utilization of batteries in devices as pacemakers^20^ or endoscopic capsules. Regarding the type of coils used, many low power applications use single-layer spiral resonators with a square or rectangular structure^26–28^. There has been some research with skin-interfaced bioelectronics powered by WPT, but these usually operate with far-field techniques^7,29,30^. In^31^, a wireless body area sensor network used to monitor several human physiological signals that use stretchable RFID passive tags is presented. In^32^, an Ultra-High Frequency (UHF) RFID fed epidermal board is used for temperature and sweat monitoring. Moreover, skin electronics powered by WPT systems can also be used to transmit sensations to the skin^33^. Regarding near-field inductive WPT for biomedical applications, most of the research is done with implantable devices^25,34,35^. Near-field WPT has the advantage of achieving higher power transmission and efficiency compared to far-field systems. Also, in terms of integration into ultrathin electronic films, near-field wireless energy harvesting is an excellent option, since only a conductive coil should be printed on the film, and there is no need for integration of rigid microchips.

However, for an efficient energy harvesting, these printed coils should fulfil a few requirements. These coils should benefit from excellent conductivity, for the best efficiency of the energy transfer. They should be stretchable, to remain functional when deformed during the natural body movement, and they should benefit from a low gauge factor, so that the applied deformation does not reduce significantly their efficiency.

For instance, as the conducting material for the tattoo circuits, the application of printed conductive polymers such as PEDOT: PSS (poly(3,4-ethylenedioxythiophene) polystyrene sulfonate)^13^, and screen printed carbon based conductors^10^ are promising for skin-interfacing electrodes. However, they are not a good option for energy harvesting due to their low electrical conductivity. Various types of conductive composites were also developed using different blends of elastic polymers and conductive micro/nanoparticles, nanowires, or nanotubes^36–38^, for stretchable electronics, but conductive composites typically suffer from the “Mullin’s effect”^39^ and degradation of the percolating network when stretched.

Electronic tattoos based on ultrathin layers of Au or Cu^7^, benefit from excellent conductivity, and low gauge factor. However, to afford strain, conductive traces are made with a horse-shoe geometry, which highly limits the number of turns that can be fitted in a fixed area. Since by increasing the number of turns in a coil it is possible to increase the quality factor and thus the maximum efficiency of the WPT system, horse-shoe/serpentine geometries limit the overall efficiency of the energy harvesting. Moreover, fabrication of this types of thin-film circuits is labour intensive and requires complex steps and setups such as metal deposition through CVD/PVD (Chemical/Physical Vapour Deposition), and posterior lithography based patterning.

Among these approaches, liquid metal (LM) – i.e. metals or metal alloys that are liquid at room temperature – are excellent options due to their fluidic compliance and high electrical conductivity. The most popular LM alloys for stretchable electronics are eutectic gallium-indium (EGaIn; 75.5 wt.% Ga and 24.5 wt.% In), and gallium-indium-tin (Galinstan; 68 wt.% Ga, 22 wt.% In and 10 wt.% Sn), which have electrical conductivities of 3.4 × 10^6^ and 3.5 × 10^6^ S/m respectively^40^. When compared with different materials for the production of stretchable electronic applications, LM-based architectures demonstrate an excellent combination of conductivity and stretchability with lower electromechanical coupling^41^. In this respect, LM alloys like EGaIn and Galinstan are well suited as conductive circuit wiring for digital electronics. However, despite rapid advances on EGaIn patterning^42–44^ and printing techniques^45^,^46^, scalable fabrication of LM based circuits is still challenging^41^. Besides, due to the liquid nature of EGaIn based LMs, they need a protection layer, which increases the thickness of the electronic tattoo film and production complexity. This works addresses these challenges by using an AgInGa printed circuit that has excellent conductivity (sheet resistance of 0.13Ω/□)^47^, with modest electromechanical coupling (gauge factor ≈1). Besides, it can be produced by simple printing techniques, and thus the geometry of the coil and the circuit can be easily customized. In addition to the efficient energy harvesting, biopotential acquisition electrodes can be printed with the same technique, and as it will be demonstrated, they provide excellent signal quality, comparable with the gold standard electrodes.

In summary, the combination of good conductivity, tolerance to strain, low gauge factor, and low skin-electrode impedance are necessary parameters for successful signal acquisition and energy harvesting. Fulfilment of these requirements along with facile fabrication technique presented in this work, pave a step toward low-cost fabrication and efficient application of these electronics tattoos for long-term, and fully wireless, battery-free and untethered monitoring of electrophysiological data.

**Figure 1:**
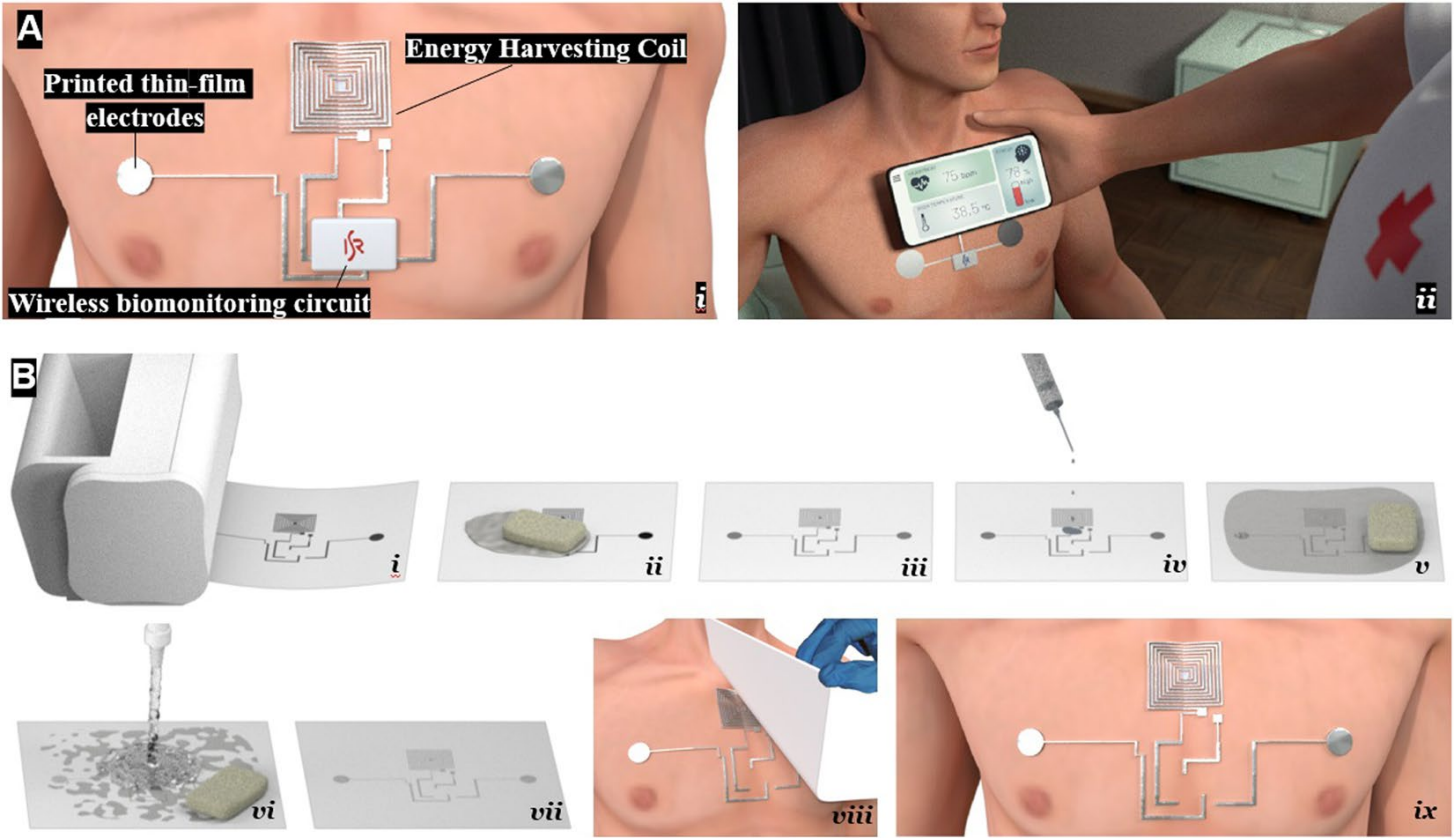
Disposable Electronic Tattoo with energy harvesting coil for fully untethered wireless ECG monitoring. (**A**) The ECG patch includes energy harvesting coil, two skin interfacing bio-potential electrodes, and a re-utilisable battery-free electronic circuit for data acquisition, processing and communication. (i) Example of the “Data-By-Demand” system. By approaching the mobile device, the electronic tattoo receives the required energy and wirelessly communicates physiological data such as heartbeat and body temperature to the same mobile device (ii). (**B**) Steps for fabrication of the printed ultrathin disposable electronic tattoo. Printing the circuit pattern using an ordinary laser printer and black toner over transfer tattoo paper (i). A silver epoxy is deposited and rubbed with a fabric (ii) rubbing is continued until the excess is removed. Ag bonds selectively to the laser printed pattern (iii). Trace amount of Liquid metal is deposited (iv), spread (v), and cleaned with a weak acetic acid solution (vi). LM wets selectively the parts with the Ag. (vii). The circuit is applied to the skin and is wet by a fabric. The backing paper is removed (viii), and the ultrathin polymer carrier with the circuit facing to the skin is transferred to the skin (ix).

In this work, we demonstrate a quickly deployable, battery-free, passive “electronic tattoo” for electrophysiological monitoring (Fig. 1). The patch, represented in Fig. 1A,B is a low cost (<1$), ultrathin (~5 µm) printed circuit that incorporates skin-interfacing electrodes and an Ag-In-Ga coil for wireless power transfer (WPT) system. This disposable circuit is interfaced with a miniature, reusable ECG circuit, that is able to acquire and transmit the heart rate. The whole process for fabrication of the patch is performed in the environment condition. There is no need for post-baking of the samples for sintering, making the fabrication step compatible with heat sensitive substrates such as paper, or ultrathin polymeric films. In addition, the process does not require photolithography or access to a clean room. The initial printing of the circuit pattern is performed by an ordinary accessible desktop laser printer, making it possible to rapidly implement custom-made circuits. The overall presented system is a “data-on-demand” system. When the caregiver approaches a scanner device to the patch, it receives the required energy to read, process and communicate the information to the scanning device via Bluetooth. This has applications in in-patient and out-patient monitoring.

## Methods

### Experimental section

Referring to Fig. 1, the overall concept relies on three main components. The battery-free patch, the electrophysiological acquisition/transmission electronics, and the scanner device composed of an energy transfer coil and the application for receiving the data. In this work, the coil and the smartphone are used separately. Such system can be used in different scenarios. This can include a data-on-request system for caregivers to check the patient’s condition with a scanner device based on a schedule. This system may be extended to a continuous monitoring system, provided that a transmission coil can be integrated into the patient’s clothing (not the focus of this article). Also, such architecture can be extended to data-on-request on implanted devices. For the case-study presented in this work for heartbeat estimation, the patch includes a receiver coil and two electrodes.

### Fabrication of the ultrathin circuit

Referring to Fig. 1B, first the pattern is printed over a temporary tattoo paper (Silhouette) using an ordinary laser printer (MFC-L2700DW; Brother) and toner (TN2320; Brother) (i), followed by coating the circuit with silver epoxy (Atom Adhesive-DUCT AD1) (ii). The silver epoxy selectively wets only where the pattern is printed (iii), but yet it is not conductive nor stretchable. Afterwards, trace amounts of eutectic Gallium-Indium (EGaIn) liquid metal are coated over the paper (iv, v). The sample is then cleaned using an (2 wt.%) aqueous solution of acetic acid (vi). In this case, EGaIn is removed from the paper, but selectively wets the circuit pattern (vii), due to the existence of the Ag Epoxy. This results in a conductive and stretchable circuit. We have previously shown a similar technique for hydroprinting these ultrathin circuits over 3D printed parts^48^, that can be used to add sensors and electronics circuits to the 3D surface of plastic parts, with application in structural electronics and novel forms of Human Machine Interfaces. Here, we extend the use of this technique for the acquisition of electrophysiological data. The reason Ag epoxy selectively wets laser printed patterns, is related to the higher adhesion between the epoxy and toner when compared to the smooth PVA layer over the tattoo paper. The PVA layer allows the adhesive to be cleaned by wiping. However, the Ag particles get trapped on the rough surface of the toner. The EGaIn then selectively wets of Ag, due to the strong adhesion between the In in the EGaIn and Ag, as previously discussed in^49^. In fact, Ag micro particles act as “docking points” for LM. The ultrathin polymeric film of the tattoo paper is ~5 µm thick. The thickness of the AgInGa layer can vary between ~2 µm if the Ag is deposited by inkjet printing, to up to 100 µm, if Ag is printed by screen printing, stencil printing or similar.

Prior to transfer to the body, the patch is covered by a thin transparent plastic coating (Plastik) as an isolator, except the electrodes, that remain uncoated. Then, as shown in Fig. 1B (viii and ix), the circuit is placed over the skin, and wet by a humid towel. Upon wetting, the water-soluble layer separates the backing paper of the tattoo paper from the carrier film, and the carrier film adheres to the human skin. The results circuit is a stretchable Ag-In-Ga conductor both for the receiver coil and the skin-interfacing electrodes. EGaIn is a non-toxic metal alloy that is liquid at room temperature and mixes with silver to form thin, highly conductive and mechanically robust circuits. These traces maintain high electrical conductivity and low electromechanical coupling (gauge factor ~1) for axial strains up to 80% and can withstand strains above 110% prior to complete electrical or mechanical failure^49^.

The low quantity of Ag and Indium deposited on each patch (<10 g for both), and the scalable fabrication method performed at the ambient conditions, allows the development of low-cost disposable patches

### ECG monitoring circuit

**Figure 2:**
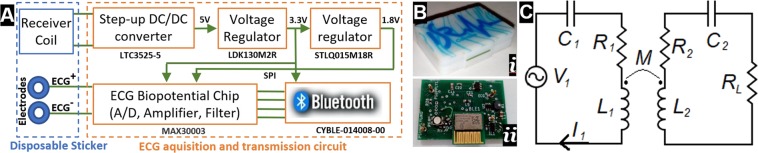
Main components of the ECG acquisition and transmission board: (**A**) scheme of the circuit; (**B**) (i) picture of the box of ECG acquisition system and (ii) of the PCB board; (**C**) WPT resonant circuit used^55^.

A miniature ECG monitoring circuit was developed for the demonstration of the application in this work. This device is composed of data acquisition, amplification, processing and communication via Bluetooth and is connected to the patch through 4 connections, two as ECG signal inputs and another two as power input (respectively “ECG−”/“ECG+” and “Receiver coil” (Fig. 2A). The two main components of this device are a Bluetooth modulus (CYBLE-014008-00) and ultra-low power, single-channel integrated biopotential chip (MAX30003) (Fig. 2B). For the case of heartbeat acquisition and communication via Bluetooth, the power consumption of the ECG circuit is ~1.6 *mW* (3.3 V, 500 µA).

### WPT circuit

A near-field (inductive) resonant circuit with series-series compensation, is shown in Fig. 2C. The circuit is composed by a power source (V1), providing a high-frequency current that feeds a transmitter coil that, through a mutual inductance M, will induce a current in the receiver coil which is connected to a load (R_L_). The coils are represented by their self-inductance (L) and intrinsic resistance (R). Then, a capacitor is connected in series with the coil, in order to tune the system to its resonant frequency, *f*_0_ = 1/(2*π*√*LC*).

In a series-series WPT system, the maximum efficiency of the power transmission to a load R_L_ is given by^25^:

1 $$\eta =\frac{{k}^{2}{Q}_{1}{Q}_{2}}{{(1+\sqrt{1+{k}^{2}{Q}_{1}{Q}_{2}})}^{2}}$$

being k, the coupling factor between the emitter and the receiver (*k* = *M*/√*L*_1_*L*_2_) and Q the quality factor of each coil (*Q* = (*ωL*)*/R*). While the coupling factor *k* will depend on the geometric dimensions of the coils and the distance between them, the quality factor will also depend on the material of the conductor (affecting the resistance R) and on the operating frequency (*ω* = *2πf*).

**Figure 3:**
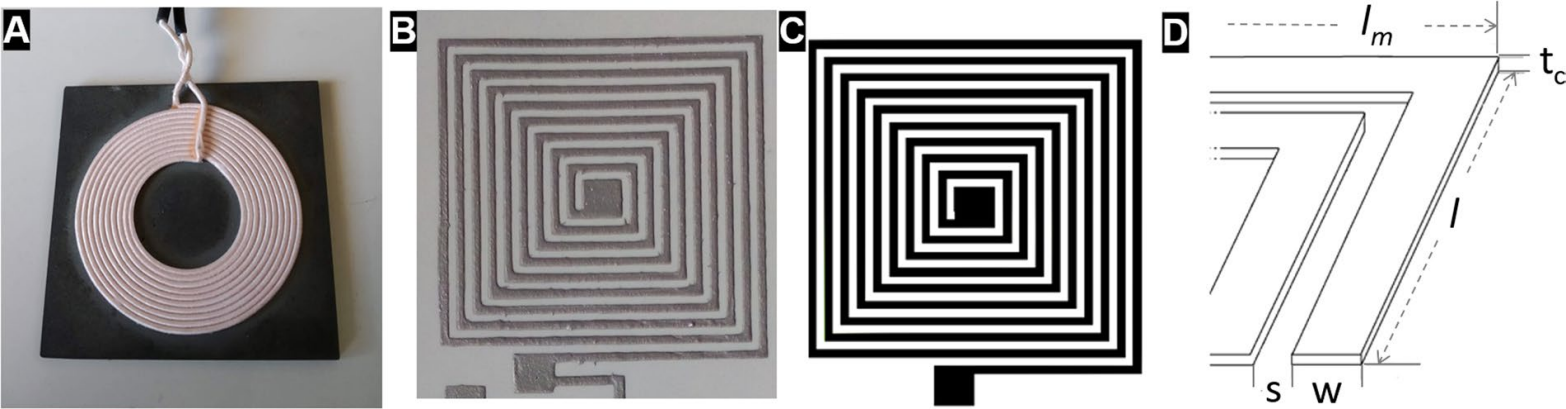
Transmitter coil (**A**), fabricated receiver coil (**B**) and receiver coil drawing (**C**) with dimensions (**D**).

In this paper, a single-layer spiral square coil was used as a receiver coil. The drawings and dimensions of the printed coil are represented in Fig. 3C,D and described in Table 1, using the same nomenclature as in^27^: length of the larger side (*l*); length of the shorter side (*l*_*m*_); space between tracks (*s*); width of the track (*w*); thickness of the track (*t*_*c*_). The final printed coil is shown in Fig. 3B.

**Table 1:** Geometric Dimensions of the Fabricated Coils.

	Dimension (mm)
Length of the larger side (*l*)	41
Length of the shorter side (*l*_*m*_)	40
Space between tracks (*s*)	1
Width of the track (*w*)	1
Thickness of the track (*t*_*c*_)	~0.05

As the transmitter coil, a coil from a mobile Qi charging pad was used (Fig. 3A). Although the increase of the operating frequency increases the maximum efficiency (Eq. 1), due to the limitations of the power source, the inductance of the transmitter coil and the capacitors commercially available the chosen resonant frequency was *f*_0_ = 495 kHz. The coil parameters were measured using an ISO-Tech LCR819 12Hz-100kHz LCR Meter at a frequency of 100 kHz. Moreover, estimating that the resistance of the conductor is expecting to increase approximately with the square root of the frequency, $\sqrt{f}$, the quality factor is estimated for the resonant frequency of 495 kHz. The characteristics of the coils, their quality factor, their mutual inductance at a distance of 0.5 mm and the added capacitors are shown in Table 2.

**Table 2:** Electric Parameters of The Transmitter and Receiver Coils.

	Transmitter	Receiver
Inductance ($\mu$ H)	6.8	2.2
Resistance ($\Omega$)	0.04	24
Added capacitance (nF)	15	47
Measured quality factor (Q) @ 100 kHz	106	0.06
Estimated quality factor (Q) @ 495 kHz	236	0.13
Mutual Inductance ($\mu$ H) (d = 0.5 mm)	3.4	

As seen in Table 2, the lower value of the quality of the receiver is due to the low thickness of the track and lower conductivity of the material used compared to copper.

## Results and discussion

### Tattoo electrodes

**Figure 4:**
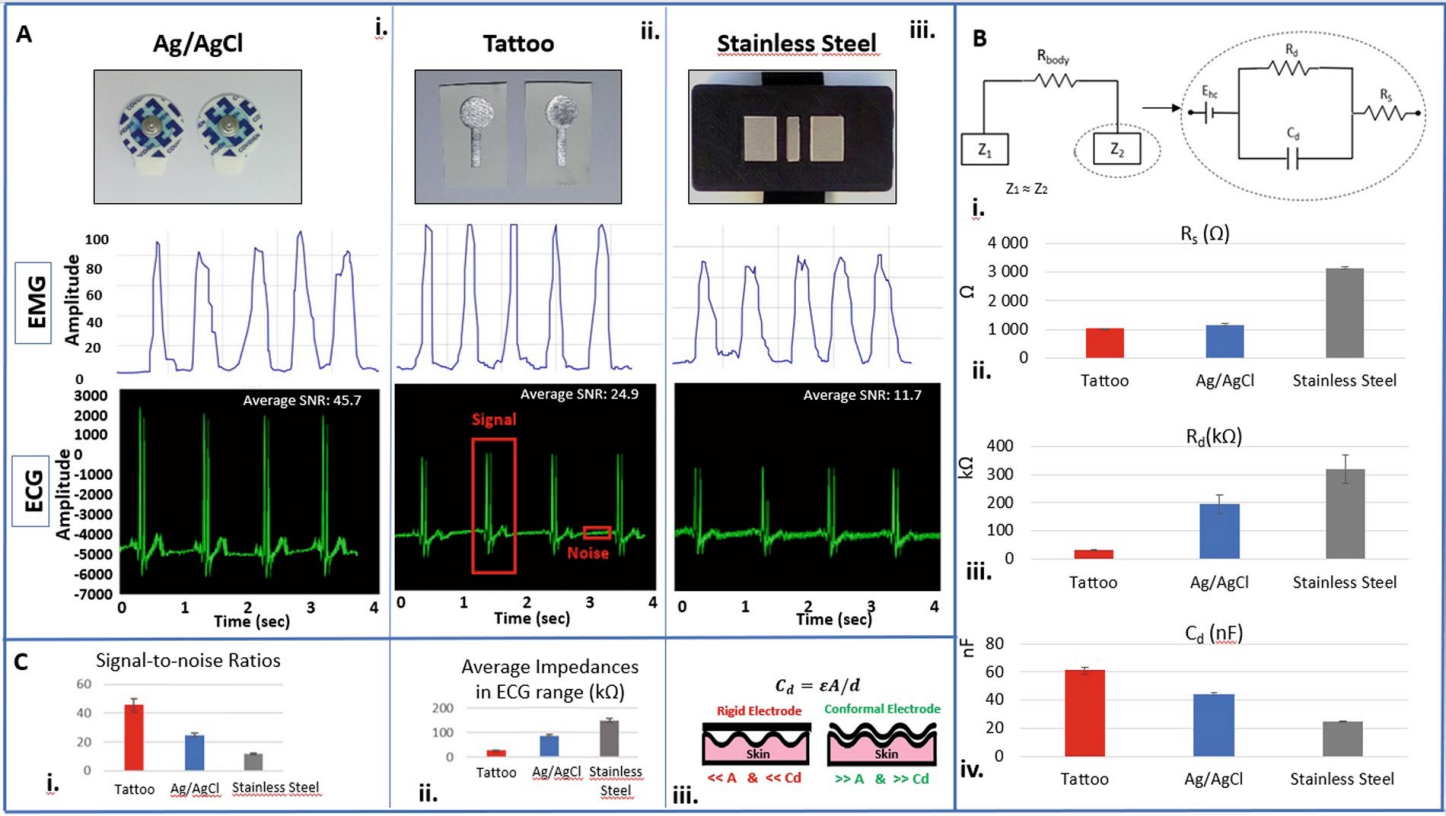
Comparison between the “tattoo electrodes”, Ag/AgCl electrodes and Stainless Steel electrodes. (**A**) Acquired signal during ECG and EMG comparison of the electrode-skin impedance for different electrodes, average and std of 5 acquisitions, for Ag/AgCl electrodes (i), Stainless Steel electrodes (ii) and “tattoo electrodes” (iii). (**B**) The model used in the analysis (i), the value of *R*_*S*_ (ii), *R*_*d*_ (iii) and *C*_*d*_ (iv). (**C**) Comparison of Signal to Noise ratio during ECG acquisition for 5 measurements (i), and the skin-electrode impedance (ii). The representation of a conformable interface vs a non-conforming interface (iii).

To analyse the suitability of the “tattoo electrodes” for the acquisition of electrophysiological data, they were compared against gold-standard dry and wet electrodes, i.e. Ag/AgCl electrodes and Stainless steel electrodes respectively. Figure 4Ai,ii,iii shows the electrodes and a snapshot of the signal acquired during EMG and ECG experiments. Figure 4Bi shows the equivalent skin-electrode interface, a model previously presented in the work of Albulbul *et al*.^50^.

In order to find out suitability of the electrodes for acquisition of electrophysiological data, we characterized the impedance between the electrode and the skin for different electrodes, using an *Agilent 4294 A* impedance analyzer. In a frequency range of 40 Hz and 100 kHz, we obtained the impedance of these electrodes in response to an alternate sinusoidal excitation electrical current. Afterwards, referring to electrical circuit model of Fig. 4Bi, we fitted a model using a least-squares non-linear fitting, according to the following equation: ${Z}_{e}={R}_{S}+\frac{{R}_{d}}{1+j2\pi f{C}_{d}{R}_{d}}$. Here, R_d_ and C_d_ are the electrode-skin interface resistance and capacitance respectively. R_S_ is the sum of the resistances of the electrode material and the electrical path between electrodes in the arm surface tissues Fig. 4Bii, iii, iv, summarizes the equivalent values of R_d_, C_d_, and R_s_ (average and standard deviation from five acquisition from the same volunteer, on the same location of the arm). As can be seen, “tattoo electrodes” benefit from a higher interface capacitance, and lower interface resistance, and therefore an overall lower skin-electrode impedance, when compared to other electrodes. The average overall skin-electrode impedance for all electrodes can be seen in Fig. 4Cii, which shows a smaller overall impedance for tattoo electrodes compared to the other electrodes, when compared to the gold standard medical grade Ag/AgCl electrodes and stainless steel dry electrodes. This can be justified by the better conformance of the thin/film electrodes to the skin, which can result in a higher interface capacitance and lower interface resistance, as seen in Fig. 4Ciii. In fact, other work also showed that electrodes deposited on ultrathin plastics benefit from a better signal quality^12^. We then compared directly the signal to noise ratio for these electrodes during five ECG measurement (Fig. 4Ci), which once more demonstrates a better SNR value for the “tattoo electrodes”, followed by the Ag/AgCl and Stainless steel electrodes.

### Power transmitted by the WPT system

In order to test the power transmission capability of the built WPT system, the power transmitted to a load was measured for several distances. A load of *R*_*L*_ = 100Ω was used as shown in the circuit of Fig. 2C, which was found out to be the optimum load for the shortest distance.

**Figure 5:**
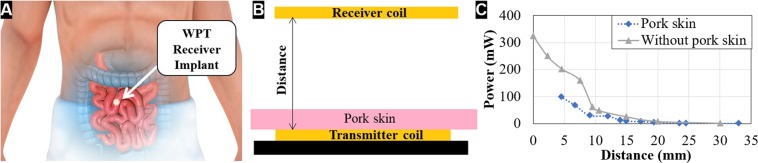
Concept for an implanted biomedical device with a near-field energy harvesting coil (**A**), schematics of the experiment performed to simulate the effect of porcine skin in between the receiver and transmitter coil (**B**) and results of the simulation comparing the power output with and without pork skin in between the coils (**C**).

Using a fixed input voltage, the voltage was measured at the terminals of the load resistance and the power was measured for different distances between the transmitter and receiver (Fig. 5C) using the following expression:

2 $${P}_{L}=\frac{{V}_{L}^{2}}{{R}_{L}}$$

It can be seen that if the coil is transferred to the skin and placed outside the body, the system can reach up to 324 mW of transmitted power. Also, considering that the ECG device is able to send the heartbeat information with 1.6 mW of consumed power, the built device could potentially work at a distance more than 20 mm from the transmitter coil. Knowing that small biomedical devices can have a power consumption that can go from 10 μW (pacemaker) to 100 mW (retinal stimulator), as referred in^51^, having a transmitted power of ~300 mW covers most of the biomedical devices.

Furthermore, in order to test the capability of the fabricated coil to transmit power in case it is implanted under the human skin, as the example in Fig. 5A shows, a piece of pork skin (source: local supermarket) with a thickness of 4.45 mm was inserted between the emitter and receiver coils, as shown in Fig. 5B. As referred in^52,53^ the pork skin has similar electric characteristics than human skin. The characteristics of the pork skin are presented in Table 3. The results were shown in Fig. 5C.

**Table 3:** Geometrical and electrical parameters of the pork skin used.

Parameter	Value
Thickness (mm)	4.4
Length × width (mm)	117.6 × 72.8
Conductivity (mS/m)	0.039

As can be seen in Fig. 5C, the power transmitted is lower in the presence of the pork skin, especially for lower distances, reaching a maximum of 100 mW. However, from a distance of 10 mm, the power transmitted to the load is approximately the same with and without the presence of skin.

**Figure 6:**
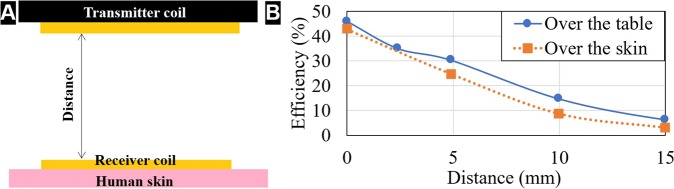
Schematics of the experiment performed to simulate the effect of human skin under the receiver coil (**A**) and results of efficiency measurements with and without human skin under the receiver coil (**B**).

Moreover, in order to test the capability of power transmission over the skin, the efficiency ($\eta )$ of the WPT system was measured over the skin as shown in Fig. 6A, according to:

3 $$\eta =\frac{{P}_{L}}{{P}_{in}}=\frac{{P}_{L}}{{V}_{1}{I}_{1}}$$

in which, *P*_*L*_ was determined as in (2) and *V*_1_ and *I*_1_ are the RMS values of the voltage and current as represented in the circuit in Fig. 2C.

Considering an input power of 0.71 W, a maximum efficiency of 46% was measured in the case the coil is over the table and 43% in the case is over the skin, as shown in Fig. 6B. Moreover, it is possible to see that the efficiency is slightly lower when the coil is placed over the skin for all the distances. Finally, using Eq. (1) and the estimated values from Table 2 to calculate the maximum efficiency of the built WPT device at the resonant frequency of 495 kHz, the value obtained was 67%.

### Case study for wireless heartbeat transmission

**Figure 7:**
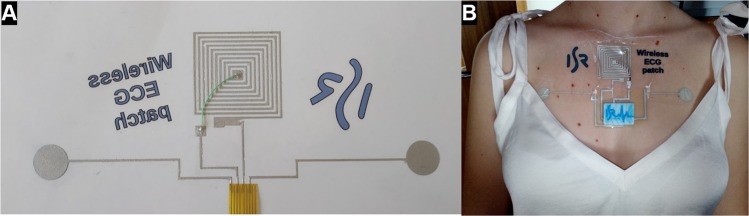
Example of the prototype of the printed electronic tattoo ready to transfer (**A**) and the full ECG monitoring circuit transferred to the human body (**B**).

After printing the patch over the temporary tattoo paper (Fig. 7A), it was transferred to the skin as shown in Fig. 7B.

Similar to temporary tattoos, the paper is soaked gradually with water until the ultrathin polymer film is transferred to the skin, while the circuit is facing to the skin. As previously mentioned, the only conductive part that interfaces the skin is the electrodes. Other parts of the circuits were previously isolated using Plastik spray. The interface between the tattoo electrode and the external circuits is made through a flexible printed circuit (FPCB) that is bonded to the printed patch using the same silver epoxy used in this work (Atom Adhesive). This FPCB is a simple copper circuit over a kapton film that allows the box to be interfaced with the patch. The transfer method is represented in Fig. 1B.

**Figure 8:**
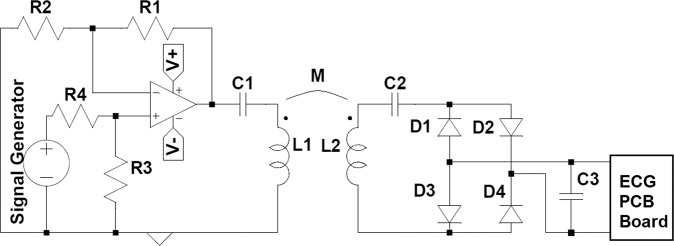
Scheme of the experimental setup built in the laboratory.

In order to feed the transmitter coil with a high-frequency current, it was connected to a Texas-Instruments OPA554 Power Op-Amp and the Op-Amp was then connected to a TTi TG330 Function Generator. The DC supply voltage of the op-amp (V+ and V− in Fig. 7) was provided by an Aim-TTi EX35RT Triple Power Supply. The scheme of the full experimental setup is represented in Fig. 8.

**Figure 9:**
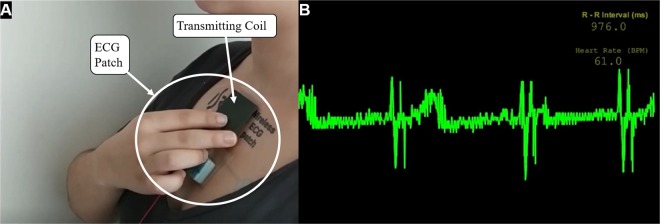
Picture of the built prototype transferred to the skin being used to transmit the ECG information (**A**) and the ECG signal received by the computer with the beats-per-minute (BPM) information (**B**).

The prototype was tested (Fig. 9A) and it was able to transmit by Bluetooth the heartbeat signal and the BPM (beats-per-minute) ratio, as it can be seen in Fig. 9B. The multimedia extension as well demonstrates the working prototype of the system. While this system is demonstrated for a “data-on-demand” system, the transferred patch may be used in a continuous monitoring mode in the future.

The 5 µm plastic layer plastic layer that carries the printed circuit bonds to the skin trough the PVA adhesive, and can stay functional for several hours. The functionality time depends on conditions of the skin, sweating, and the location of application on the body.

In summary, this works demonstrates a fully untethered, wireless (data and energy), and battery-free electronic tattoo, that is able to acquire and transmit electrophysiological data, such as heart rate to an external device. To do so, materials and methods for the printing of thin-film stretchable electronics that are efficient both in terms of energy harvesting, and signal acquisition were demonstrated. The low skin-electrode impedance for this type of bi-phasic AgInGa conductors, is an important factor in the excellent SNR value of the signals acquired by these electronic tattoos.

### Multimedia extension

The multimedia extension supplementary video shows the developed system functioning. When approaching the transmitter coil to the transferred coil, the ECG patch starts functioning and transmit the bpm of the volunteer to a computer application via Bluetooth, as shown as well in Fig. 9.

## Conclusions and Future Work

In this paper, we demonstrate a low-cost battery-free rapidly deployable and disposable patch for electrophysiological monitoring. This was performed by printing a stretchable AgInGa circuit over a temporary tattoo paper that is then transferred to a volunteer’s chest. The disposable patch BOM cost is less than 1$US, and can be rapidly customized with the presented printing technique. We showed that the electrodes printed with this method provide low skin-electrode impedance and excellent signal to noise ratio. The patch is then interfaced with a battery-free circuit for data acquisition, processing and communication via Bluetooth. We demonstrate a “data-on-demand” system that transmits the patient’s heart rate, every time the mobile device approaches the patient´s chest. In order to transfer the required energy, we relied on a wireless power transfer system. We showed that the WPT device can provide ~300 mW of power when transferred over the skin, which is in an acceptable range for many biomedical devices and applications.

Skin-interfacing electrodes utilized in this work are based on EGaIn liquid metal. A study^54^ on cytotoxicity tests of EGaIn liquid metal concluded that EGaIn is reasonably safe to use in an aqueous environment; however, it should be cautiously handled when any mechanical agitation is applied. Yet, further analysis should be performed to ensure full biocompatibility.

In order to test the feasibility of the system for implantable devices, we tested the energy transfer under the porcine skin. Results show that although the power transferred was lower compared to the case where the receiver is over the skin, a maximum of 100 mW could be achieved.

Future works include development and validation of other patches for biomonitoring through measurement of bio-potentials, and other electrophysiological data such as skin conductivity, body temperature, and breath rate. The overall architecture of these patches will be kept similar to this work, but will differ in terms of the design of the patch and electronics circuits. Moreover, more tests will be done considering human skin instead of pork skin. In addition, the current work will be further improved by designing and printing of more efficient energy transfer devices. To do so, methods for higher resolution printing of stretchable circuits are being studied, that would allow increasing the number of the coil loops in a fixed area, and fabrication of multi-layer coils.

## Supplementary information

Supplementary video.

## References

[1] Haahr, R. G., Duun, S., Thomsen, E. V., Hoppe, K. & Branebjerg, J. A wearable “electronic patch” for wireless continuous monitoring of chronically diseased patients. In *2008 5th International Summer School and Symposium on Medical Devices and Biosensors*, 66–70, 10.1109/ISSMDBS.2008.4575018 (IEEE, 2008).

[2] Wu, C.-C. *et al*. A pliable and batteryless real-time ECG monitoring system-in-a-patch. in *VLSI Design, Automation and Test(VLSI-DAT)* 1–4, 10.1109/VLSI-DAT.2015.7114521 (IEEE, 2015).

[3] Akalin Acar Z, Acar CE, Makeig S (2016). Simultaneous head tissue conductivity and EEG source location estimation. Neuroimage.

[4] Tavakoli, M., Benussi, C. & Lourenco, J. L. Single channel surface EMG control of advanced prosthetic hands: A simple, low cost and efficient approach. *Expert Syst. Appl*. **79** (2017).

[5] Tavakoli M, Benussi C, Alhais Lopes P, Osorio LB, de Almeida AT (2018). Robust hand gesture recognition with a double channel surface EMG wearable armband and SVM classifier. Biomed. Signal Process. Control.

[6] Maragliulo, S., Lopes, P. F. A., Osorio, L. B., de Almeida, A. T. & Tavakoli, M. Foot Gesture Recognition through Dual Channel Wearable EMG System. *IEEE Sens. J*., 10.1109/jsen.2019.2931715 (2019).

[7] Kim D-H (2011). Epidermal Electronics. Science (80-.)..

[8] Gong S (2015). Tattoolike Polyaniline Microparticle-Doped Gold Nanowire Patches as Highly Durable Wearable Sensors. ACS Appl. Mater. Interfaces.

[9] Zucca A (2015). Tattoo Conductive Polymer Nanosheets for Skin-Contact Applications. Adv. Healthc. Mater..

[10] Bareket, L. *et al*. Temporary-tattoo for long-term high fidelity biopotential recordings. *Sci. Rep*. **6** (2016). 10.1038/srep25727 PMC4864418 27169387

[11] Ferrari, L. M. *et al*. Ultraconformable Temporary Tattoo Electrodes for Electrophysiology. *Adv. Sci*., 10.1002/advs.201700771 (2018). 10.1002/advs.201700771 PMC5867059 29593975

[12] Jeong, J. W. *et al*. Materials and optimized designs for human-machine interfaces via epidermal electronics. *Adv. Mater*., 10.1002/adma.201301921 (2013). 10.1002/adma.201301921 24327417

[13] Greco F, Zucca A, Taccola S, Mazzolai B, Mattoli V (2013). Patterned free-standing conductive nanofilms for ultraconformable circuits and smart interfaces. ACS Appl. Mater. Interfaces.

[14] Kabiri Ameri, S. *et al*. Graphene Electronic Tattoo Sensors. *ACS Nano*, 10.1021/acsnano.7b02182 (2017). 10.1021/acsnano.7b02182 28719739

[15] Ha, T. *et al*. A Chest-Laminated Ultrathin and Stretchable E-Tattoo for the Measurement of Electrocardiogram, Seismocardiogram, and Cardiac Time Intervals. *Adv. Sci*., 10.1002/advs.201900290 (2019). 10.1002/advs.201900290 PMC6662084 31380208

[16] Shustak, S. *et al*. Home monitoring of sleep with a temporary-tattoo EEG, EOG and EMG electrode array: A feasibility study. *J. Neural Eng*., 10.1088/1741-2552/aafa05 (2019). 10.1088/1741-2552/aafa05 30566912

[17] Jia, W. *et al*. *Electrochemical Tattoo Biosensors for Real-Time Noninvasive Lactate Monitoring in Human Perspiration*. (2013). 10.1021/ac401573r 23815621

[18] Liu C, Guo Y-X, Sun H, Xiao S (2014). Design and Safety Considerations of an Implantable Rectenna for Far-Field Wireless Power Transfer. IEEE Trans. Antennas Propag..

[19] Chow EY (2011). Wireless Powering and the Study of RF Propagation Through Ocular Tissue for Development of Implantable Sensors. IEEE Trans. Antennas Propag..

[20] Campi T (2016). Wireless Power Transfer Charging System for AIMDs and Pacemakers. IEEE Trans. Microw. Theory Tech..

[21] Kim, J.-D., Sun, C. & Suh, I.-S. A proposal on wireless power transfer for medical implantable applications based on reviews. In *2014 IEEE Wireless Power Transfer Conference* 166–169, 10.1109/WPT.2014.6839592 (IEEE, 2014).

[22] Tianjia Sun (2012). A Two-Hop Wireless Power Transfer System With an Efficiency-Enhanced Power Receiver for Motion-Free Capsule Endoscopy Inspection. IEEE Trans. Biomed. Eng..

[23] Ke Q, Luo W, Yan G, Yang K (2016). Analytical Model and Optimized Design of Power Transmitting Coil for Inductively Coupled Endoscope Robot. IEEE Trans. Biomed. Eng..

[24] Manoufali M, Bialkowski K, Mohammed BJ, Mills PC, Abbosh A (2018). Near-Field Inductive-Coupling Link to Power a Three-Dimensional Millimeter-Size Antenna for Brain Implantable Medical Devices. IEEE Trans. Biomed. Eng..

[25] Xue RF, Cheng KW, Je M (2013). High-Efficiency Wireless Power Transfer for Biomedical Implants by Optimal Resonant Load Transformation. IEEE Trans. Circuits Syst. I, Reg. Pap..

[26] Alberto, J., Puccetti, G., Grandi, G., Reggiani, U. & Sandrolini, L. Experimental study on the termination impedance effects of a resonator array for inductive power transfer in the hundred {kHz} range. in *Proc. 2015 IEEE Wireless Power Transfer Conf*. (WPTC 2015) 1–4, 10.1109/WPT.2015.7139136 (2015).

[27] Alberto, J., Puccetti, G., Reggiani, U., Sandrolini, L. & Tacchini, A. Multilayer Flat Spiral Resonators for Low Frequency Wireless Power Transfer. in 2018 IEEE-APS Topical Conference on Antennas and Propagation in Wireless Communications (APWC) 873–876, 10.1109/APWC.2018.8503768 (2018).

[28] Alberto J, Reggiani U, Sandrolini L (2016). Magnetic near field from an inductive power transfer system using an array of coupled resonators. 2016 Asia-Pacific Int. Symp. on Electromagn. Compat. (APEMC).

[29] García Núñez C, Manjakkal L, Dahiya R (2019). Energy autonomous electronic skin. npj Flex. Electron..

[30] Huang X (2016). Epidermal radio frequency electronics for wireless power transfer. Microsystems Nanoeng..

[31] Niu S (2019). A wireless body area sensor network based on stretchable passive tags. Nat. Electron..

[32] Miozzi C, Nappi S, Amendola S, Occhiuzzi C, Marrocco G (2019). A General-Purpose Configurable RFID Epidermal Board with a Two-Way Discrete Impedance Tuning. IEEE Antennas Wirel. Propag. Lett..

[33] Yu, X. *et al*. Skin-integrated wireless haptic interfaces for virtual and augmented reality. *Nature* **575** (2019). 10.1038/s41586-019-1687-0 31748722

[34] Ho JS (2014). Wireless power transfer to deep-tissue microimplants. Proc. Natl. Acad. Sci. USA.

[35] Kim S, Ho JS, Poon ASY (2012). Wireless Power Transfer to Miniature Implants: Transmitter Optimization. IEEE Trans. Antennas Propag..

[36] Larmagnac A, Eggenberger S, Janossy H, Vörös J (2014). Stretchable electronics based on Ag-PDMS composites. Sci. Rep..

[37] Tavakoli, M. *et al*. Carbon doped PDMS: Conductance stability over time and implications for additive manufacturing of stretchable electronics. *J. Micromechanics Microengineering* **27** (2017).

[38] Choi S, Han SI, Kim D, Hyeon T, Kim DH (2019). High-performance stretchable conductive nanocomposites: Materials, processes, and device applications. Chemical Society Reviews.

[39] Fernandes, D. F., Majidi, C. & Tavakoli, M. Digital Printing of Stretchable Electronics: A Review. *J. Mater. Chem. C*, 10.1039/c9tc04246f (2019).

[40] Wang, L. & Liu, J. Liquid metal inks for flexible electronics and 3D printing: A review. In *ASME International Mechanical Engineering Congress and Exposition, Proceedings (IMECE) 2B*, (American Society of Mechanical Engineers (ASME) 2014).

[41] Fernandes, D., Majidi, C. & Tavakoli, M. Digitally Printed Stretchable Electronics: A Review. *J. off Mater. Chem. C* In Press (2019).

[42] Marques, D. G., Lopes, P. A., Almeida, A. T. de, C Majidi & Tavakoli, M. Reliable interfaces for EGaIn multi-layer stretchable circuits and microelectronics. *Lab a Chip* (In Press. 19, 897–906 (2019). 10.1039/c8lc01093e 30724280

[43] Lu T, Markvicka EJ, Jin Y, Majidi C (2017). Soft-Matter Printed Circuit Board with UV Laser Micropatterning. ACS Appl. Mater. Interfaces.

[44] Rocha, R., Lopes, P., de Almeida, A. T., Tavakoli, M. & Majidi, C. Soft-matter sensor for proximity, tactile and pressure detection. In *2017 IEEE/RSJ International Conference on Intelligent Robots and Systems* (IROS) 3734–3738, 10.1109/IROS.2017.8206222 (2017).

[45] Yoon, Y., Kim, S., Kim, D., Kauh, S. K. & Lee, J. Four Degrees-of-Freedom Direct Writing of Liquid Metal Patterns on Uneven Surfaces. *Advanced Materials Technologies*, 10.1002/admt.201800379 (2018).

[46] Boley JW, White EL, Chiu GTC, Kramer RK (2014). Direct writing of gallium-indium alloy for stretchable electronics. Adv. Funct. Mater..

[47] Lopes PA, Paisana H, Almeida AT, Majidi C, Tavakoli M (2018). Hydroprinted Electronics: Ultrathin Stretchable Ag-In-Ga E-Skin for Bioelectronics & Human-Machine Interaction. ACS Appl. Mater. Interfaces.

[48] Lopes, P. A., Paisana, H., De Almeida, A. T., Majidi, C. & Tavakoli, M. Hydroprinted Electronics: Ultrathin Stretchable Ag-In-Ga E-Skin for Bioelectronics and Human-Machine Interaction. *ACS Appl. Mater. Interfaces*, 10.1021/acsami.8b13257 (2018). 10.1021/acsami.8b13257 30338978

[49] Tavakoli M (2018). EGaIn-Assisted Room-Temperature Sintering of Silver Nanoparticles for Stretchable, Inkjet-Printed, Thin-Film Electronics. Adv. Mater..

[50] Albulbul A (2016). Evaluating Major Electrode Types for Idle Biological Signal Measurements for Modern Medical Technology. Bioengineering.

[51] Romero, E. Powering Biomedical Devices. *Powering Biomedical Devices*, 10.1016/C2012-0-06126-1 (2013).

[52] Vallejo M, Recas J, del Valle P, Ayala J (2013). Accurate Human Tissue Characterization for Energy-Efficient Wireless On-Body Communications. Sensors.

[53] Karacolak T, Cooper R, Unlu ES, Topsakal E (2012). Dielectric Properties of Porcine Skin Tissue and *In Vivo* Testing of Implantable Antennas Using Pigs as Model Animals. IEEE Antennas Wirel. Propag. Lett..

[54] Kim, J. H., Kim, S., So, J. H., Kim, K. & Koo, H. J. Cytotoxicity of Gallium-Indium Liquid Metal in an Aqueous Environment. *ACS Appl. Mater. Interfaces*, 10.1021/acsami.8b02320 (2018). 10.1021/acsami.8b02320 29715000

[55] Li S, Mi CC (2015). Wireless Power Transfer for Electric Vehicle Applications. IEEE J. Emerg. Sel. Top. Power Electron..

